# Supplementary material for: Identification and Functional Validation of Two Novel Antioxidant Peptides in Saffron
Source: Antioxidants (Basel). 2024 Mar 20;13(3):378. doi: 10.3390/antiox13030378 (PMC10967730; doi:10.3390/antiox13030378)
Supplement: Supplementary file 1 [file antioxidants-13-00378-s001.zip › antioxidants-2897486-supplementarya/Supplementary Material/MS and HPLC information of active peptides/DGGSDYLGK-HPLC.pdf]

Sample Name :DK
Sample ID :C982M029G0-1
Time Processed :23:22:12
Month-Day-Year Processed :04/30/2023

Pump A : 0.065% trifluoroacetic in 100% water (v/v)
Pump B : 0.05% trifluoroacetic in 100% acetonitrile (v/v)
Total Flow:1 ml/min
Wavelength:220 nm
<<LC Time Program>>

Time	Module	Command	Value
0.01	Pumps	B.Conc	5
25.00	Pumps	B.Conc	65
25.01	Pumps	B.Conc	95
27.00	Pumps	B.Conc	95
27.01	Pumps	B.Conc	5
35.00	Pumps	B.Conc	5
35.01	Controller	Stop	

<<Column Performance>>
<Detector A>
Column :Inertsil ODS-SP 4.6 x 250 mm
Equipment: GK11010011

<Chromatogram>

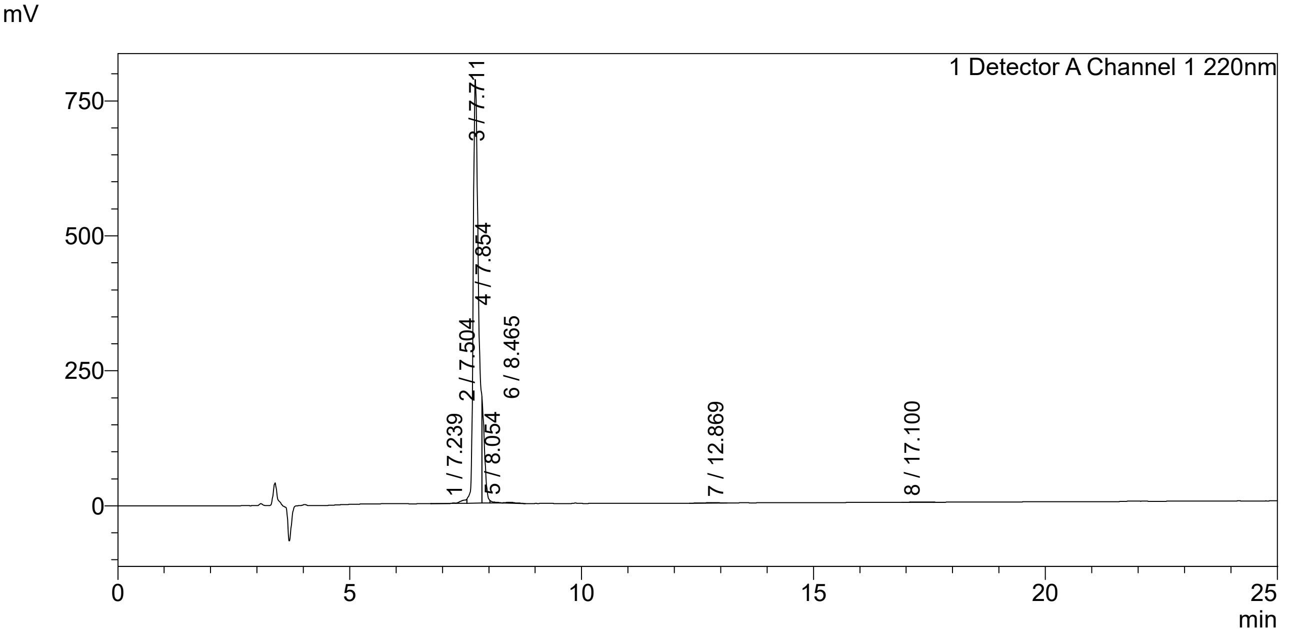

<Peak Table>

Detector A Channel 1 220nm

Peak#	Ret. Time	Area	Height	Area%
1	7.239	178	835	0.003
2	7.504	58750	6150	0.841
3	7.711	6154295	769018	88.109
4	7.854	706665	203260	10.117
5	8.054	20467	3437	0.293
6	8.465	19972	1495	0.286
7	12.869	14258	719	0.204
8	17.100	10253	335	0.147
Total		6984838	985248	100.000
