# Supplementary material for: Identification and Functional Validation of Two Novel Antioxidant Peptides in Saffron
Source: Antioxidants (Basel). 2024 Mar 20;13(3):378. doi: 10.3390/antiox13030378 (PMC10967730; doi:10.3390/antiox13030378)
Supplement: Supplementary file 1 [file antioxidants-13-00378-s001.zip › antioxidants-2897486-supplementarya/Supplementary Material/MS and HPLC information of active peptides/DGGSDYLGK-MS.pdf]

Mass Spectrum

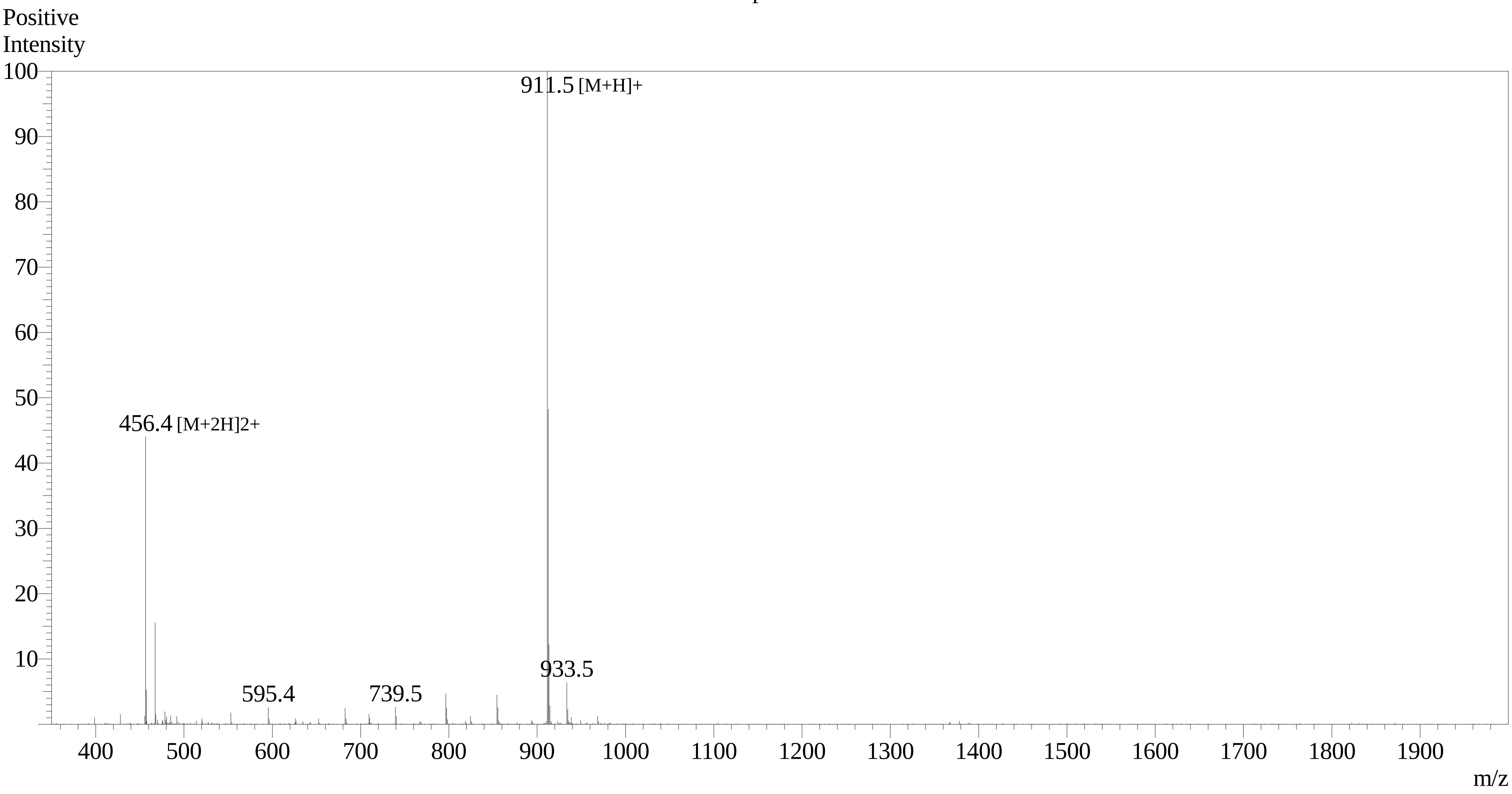

Sample Information
Month-Day Processed : 04/30/23
Time Processed : 19:59:45
Injection Volume : 0.3
Sample Name : DK
Sample ID : C982M029G0-1
Theoretical MW : 910.93
Observed MW : 910.5

Interface :ESI
Nebulizing Gas Flow :1.5L/min
CDL Temp :250
Block Temp :200

Equipment : ZJ21010035
Interface Bias : +4.5 kV
Drying Gas Flow :5 L/min
T.Flow :0.2 ml/min
B.conc :50%H2O/50%MeOH
