# Supplementary material for: Identification and Functional Validation of Two Novel Antioxidant Peptides in Saffron
Source: Antioxidants (Basel). 2024 Mar 20;13(3):378. doi: 10.3390/antiox13030378 (PMC10967730; doi:10.3390/antiox13030378)
Supplement: Supplementary file 1 [file antioxidants-13-00378-s001.zip › antioxidants-2897486-supplementarya/Supplementary Material/MS and HPLC information of active peptides/VDPYFNK-HPLC.pdf]

Sample Name :VK
Sample ID :C982M029G0-3
Time Processed :20:24:06
Month-Day-Year Processed :05/02/2023

Pump A : 0.065% trifluoroacetic in 100% water (v/v)
Pump B : 0.05% trifluoroacetic in 100% acetonitrile (v/v)
Total Flow:1 ml/min
Wavelength:220 nm
<<LC Time Program>>

Time	Module	Command	Value
0.01	Pumps	B.Conc	5
25.00	Pumps	B.Conc	65
25.01	Pumps	B.Conc	95
27.00	Pumps	B.Conc	95
27.01	Pumps	B.Conc	5
35.00	Pumps	B.Conc	5
35.01	Controller	Stop	

<<Column Performance>>
<Detector A>
Column :Inertsil ODS-SP 4.6 x 250 mm
Equipment: ZJ20010140

<Chromatogram>

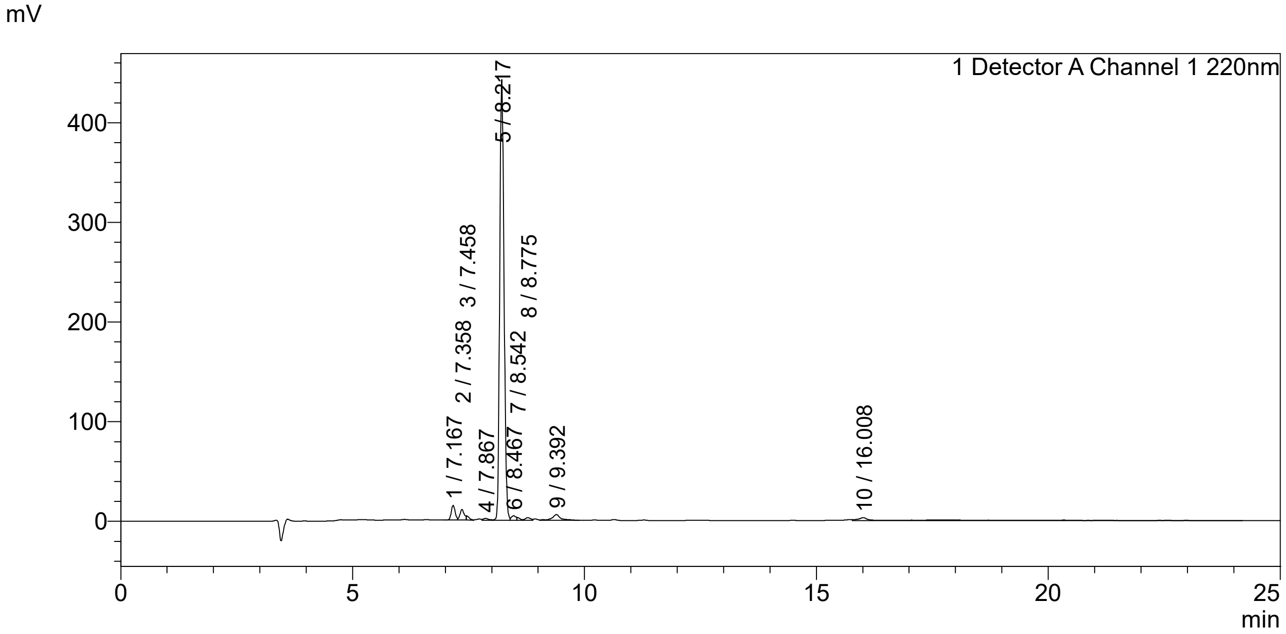

<Peak Table>

Detector A Channel 1 220nm

Peak#	Ret. Time	Area	Height	Area%
1	7.167	87268	14754	2.605
2	7.358	69273	10667	2.068
3	7.458	18562	4181	0.554
4	7.867	14151	1873	0.422
5	8.217	2843158	442271	84.858
6	8.467	30297	4481	0.904
7	8.542	12620	2968	0.377
8	8.775	21609	2659	0.645
9	9.392	77937	5865	2.326
10	16.008	175628	3057	5.242
Total		3350504	492774	100.000
