# Supplementary material for: Identification and Functional Validation of Two Novel Antioxidant Peptides in Saffron
Source: Antioxidants (Basel). 2024 Mar 20;13(3):378. doi: 10.3390/antiox13030378 (PMC10967730; doi:10.3390/antiox13030378)
Supplement: Supplementary file 1 [file antioxidants-13-00378-s001.zip › antioxidants-2897486-supplementarya/Supplementary Material/MS and HPLC information of active peptides/VDPYFNK-MS.pdf]

Mass Spectrum

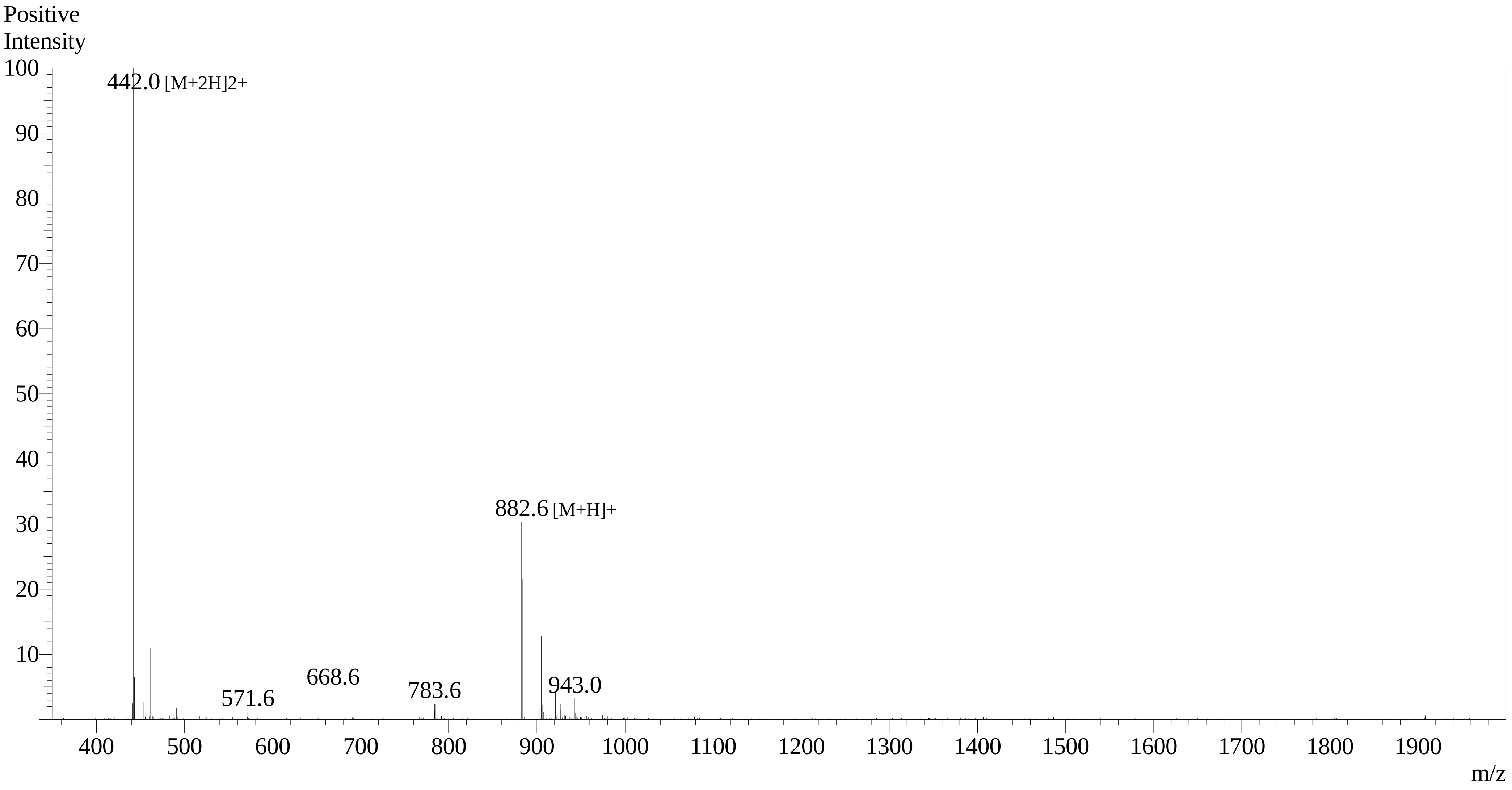

Sample Information

Month-Day Processed :	05/02/23
Time Processed :	19:02:41
Injection Volume :	0.1
Sample Name :	VK
Sample ID :	C982M029G0-3
Theoretical MW :	881.98
Observed MW :	882.0

Interface	:ESI
Nebulizing Gas Flow	:1.5L/min
CDL Temp	:250
Block Temp	:200

Equipment	:ZJ22010150
Interface Bias	: +4.5 kV
Drying Gas Flow	:5 L/min
T.Flow	:0.2 ml/min
B.conc	:50%H2O/50%MeOH
