# Supplementary material for: Identification and Functional Validation of Two Novel Antioxidant Peptides in Saffron
Source: Antioxidants (Basel). 2024 Mar 20;13(3):378. doi: 10.3390/antiox13030378 (PMC10967730; doi:10.3390/antiox13030378)
Supplement: Supplementary file 1 [file antioxidants-13-00378-s001.zip › antioxidants-2897486-supplementarya/Supplementary Material/Supplementary Figure.pdf]

Identification and Functional Validation of Two Novel Antioxidant Peptides in Saffron

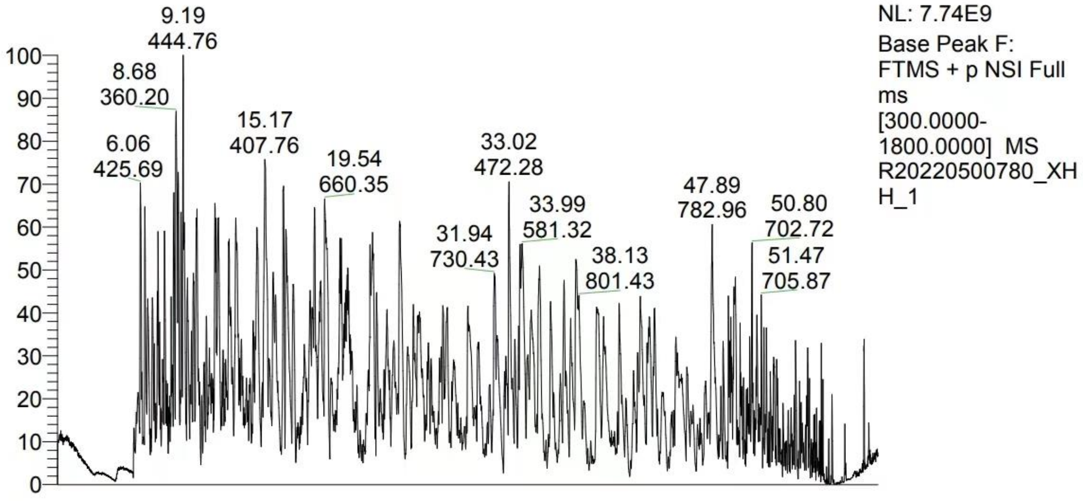

Figure S1. Saffron Protein mass spectrometry

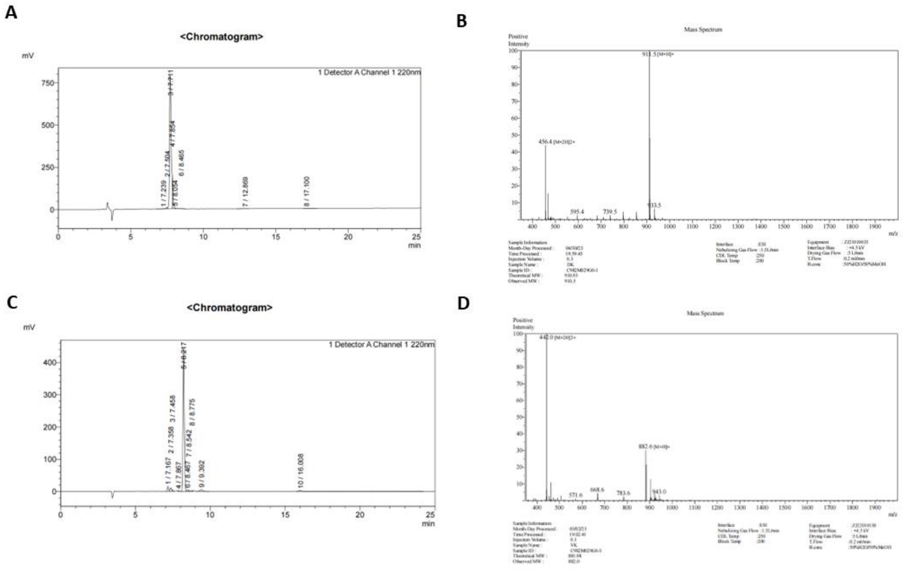

Figure S2. (A-B) Chromatographic diagram (A) and mass-spectrogram (B) of DGGSDYLGK; (C-D) Chromatographic diagram (C) and mass-spectrogram (D) of VDPYFNK
